# *Cordyceps sinensis* relieves non-small cell lung cancer by inhibiting the MAPK pathway

**DOI:** 10.1186/s13020-024-00895-0

**Published:** 2024-03-25

**Authors:** Tianming Lu, Lirun Zhou, Zheng Chu, Yang Song, Qixin Wang, Minghong Zhao, Chuanhao Dai, Lin Chen, Guangqing Cheng, Jigang Wang, Qiuyan Guo

**Affiliations:** 1 State Key Laboratory for Quality Ensurance and Sustainable Use of Dao-di Herbs, Artemisinin Research Center, and Institute of Chinese Materia Medica, China Academy of Chinese Medical Sciences, Beijing, 100700 China; 2 Department of Oncology, The Affiliated Hospital of Southwest Medical University, Luzhou, 646000 Sichuan China

**Keywords:** *Cordyceps Sinensis*, NSCLC, MAPK pathway, Transcriptomics, Proteomics

## Abstract

**Objective:**

To determine the pharmacodynamic mechanism underlying *Cordyceps sinensis* relief in a murine model of non-small cell lung cancer (NSCLC).

**Methods:**

We created a murine model of NSCLC and studied the potential molecular mechanism by which *C. sinensis* relieved NSCLC using a combination of transcriptomics, proteomics, and experimental validation.

**Results:**

*C. sinensis* markedly suppressed the fluorescence values in mice with NSCLC, improved the pathologic morphology of lung tissue, ameliorated inflammatory cytokines (tumor necrosis factor-alpha, interleukin-6, interleukin-10, and the oxidative stress indicators superoxide dismutase, malondialdehyde, and glutathione peroxidase). Transcriptomics results showed that the therapeutic effect of *C. sinensis* was primarily involved in the differentiation and activation of T cells. Based on the proteomic results, *C. sinensis* likely exerted a protective effect by recruiting immune cells and suppressing tumor cell proliferation via the MAPK pathway. Finally, the experimental validation results indicated that *C. sinensis* significantly decreased the VEGF and Ki67 expression, downregulated RhoA, Raf-1, and c-fos expression, which are related to cell migration and invasion, increased the serum concentration of hematopoietic factors (EPO and GM-CSF), and improved the percentage of immune cells (natural killer cells, dendritic cells, and CD4^+^ and CD8^+^ lymphocytes), which enhanced immune function.

**Conclusions:**

Based on our preclinical study, *C. sinensis* was shown to exert a protective effect on NSCLC, primarily by inhibiting the MAPK pathway.

**Supplementary Information:**

The online version contains supplementary material available at 10.1186/s13020-024-00895-0.

## Introduction

Its high incidence and mortality rates mean that lung cancer is ranked first among cancer types; there are two main types, small cell lung cancer (SCLC) and non-small cell lung cancer (NSCLC), with the latter accounting for 80-85% of lung cancer cases [1, 2]. There were 715,000 deaths attributed to lung cancer in China in 2020 [3, 4]. The drugs currently used for the treatment of NSCLC regulate the immune response, target specific molecules, or are directly cytotoxic [5, 6]. Blockade of programmed cell death (PD)-1 has been extensively used as a standard treatment for NSCLC, but PD-1 blockade has been associated with immune-related adverse events, including pneumonitis, dermatologic adverse events, and gastrointestinal toxicity [7–10]. Natural products from traditional Chinese medicine can be used as multi-targeted cancer therapies, which is a current trend in the treatment of NSCLC [11, 12], such as cordycepin’s anticancer effect interacting with and activating AMP-activated protein kinase (AMPK) [13], Emodin inhibited NSCLC proliferation by decreasing the expression of sPLA2-IIa and NF-κB pathways and suppressed mTOR and AKT and activated the AMPK pathway [14].

Vascular endothelial growth factor (VEGF) is a key regulator of NSCLC development via inhibition of immune cell differentiation, thereby reducing infiltration and promoting immune tumor cell escape [15]. *Cordyceps sinensis*, the content of the Bailing capsule, has anti-tumor, enhanced immunity, resistance to oxidation, fibrosis, viruses, and inflammation [16]. Previous studies showed that *C. sinensis* has therapeutic effects on chronic lung and kidney diseases, such as chronic obstructive pulmonary disease, which can improve lung function, arterial blood gas indices, and exercise tolerance [17, 18]. As reported, *C. sinensis*, as an immunosuppressive agent, inhibits the function of antigen-presenting cells, which leads to a state involving a low immune response and affects the ability of dendritic cells (DCs) to stimulate proliferation, then alleviates the toxic and side effects of chemotherapy [19]. Recently, anti-PD-1 immunotherapy has caused more and more attention in the treatments of NSCLC [20, 21]. In addition, combination therapy is a commonly used method for cancer treatment [22]. Thus, in this study, we observed the effect of the single application of *C. sinensis* as well as its combination therapy with PD-1inbihitor.

Therefore, the pharmacodynamic characteristics were evaluated and the mechanism underlying *C. sinensis* on mice with NSCLC was investigated using a combination of transcriptomics, proteomics, and experimental validation.

## Materials and methods

### Drugs and reagents

*C. sinensis* (Bailing capsule, BL, SFDA approval number: Z10910036, batch No. 2,106,302 A) was provided by Zhong Mei Hua Dong Pharmaceutical Co. LTD (Hangzhou, China), and its main ingredient is fermented *Cordyceps* powder. The fresh natural *C. sinensis* was collected, dried in cool, well-ventilated conditions and then fermented under certain conditions. The fermented and qualified powder is packaged as medicine. Based on UHPLC-LTQ-Orbitrap MS, qualitative analysis of Bailing Capsules obtained a total of 58 compounds, including 17 nucleosides, 15 amino acids, 5 purine alkaloids, 4 pyrimidine alkaloids, 8 organic acids, 2 nucleotides, 2 sugar alcohols and 5 other compounds. 12 compounds (D-mannitol, cordycepin, tyrosine, uridine, arginine, histidine, inosine, adenosine, guanosine, 5ʹ-guanylic acid, 5ʹ-adenosine, L-lysine) were determined by comparison with the standard substances (Additional file 1, Figure S1). Tislelizumab injection was purchased from BeiGene Ltd. (Beijing, China). Saline (0.9%) was provided by Shandong Kelun Pharmaceutical Co., Ltd. (B22082402A; Shandong, China). Matrigengel matrix was obtained from Shanghai Nova Medical Technology Co., Ltd. (0827045; Shanghai, China).

## NSCLC mouse model construction

Lewis lung carcinoma (LLC) cells obtained from Zhong Qiao Xin Zhou Biotechnology (LZQ0009; Shanghai, China) were incubated in DMEM medium (containing 10% fetal bovine serum and 1% penicillin) in 5% CO_2_ at 37 ℃. To generate the NSCLC mouse model, LLC cells (80–90% growth) were subjected to trypsin digestion and were then collected and washed with phosphate-buffered saline (PBS), according to a previous study [23]. Cells were added to Matrigel at a 1:1 ratio after centrifugation, then 1 million cells were injected into each mouse.

C57BL/6J mice (*n* = 36, weight 20 ± 3 g; Beijing Vital River Laboratory Animal Technology, Beijing, China) were acclimatized under laboratory conditions for 1 week. The temperature of the feeding room was 25 ± 2 ℃, relative humidity of 50–60%, and a 12-h light, 12-h dark cycle. The mice had free access to food and water. Approval from the Care and Use of Laboratory Animals of China Academy of Chinese Medical Sciences (No. 2022B139) was obtained for the animal experiments.

After 7 days of adaptive feeding, 36 male C57BL/6J mice were randomly assigned to six different groups (*n* = 6 each). Each mouse was anesthetized and a prepared Matrigel mixture was injected vertically into the left lung at a depth of approximately 4 mm. The injection remained for 20 s. The mice were returned to the cage after awakening from anesthesia. The same procedure was performed in the control group, but no Matrigel mixture was injected.

### Grouping and administration

The LLC cells used for modeling were stably transfected with luciferase, which emitted fluorescence under substrate excitation. According to a previous study [23], IVIS lumina Series III (PerkinElmer, Waltham, MA, USA) was used to detect the inoculation results on the 5th day. Each mouse was given an intraperitoneal injection of 150 µL of D-luciferin (Luc-1G, 115144-35-9; Gold Bio-Technology, St. Louis, MO, USA) and observed for 15 min. Then, fluorescent pictures were obtained with a small animal imager and counted. The mice that were successfully modeled were further divided into the following 5 groups (*n* = 6 each), including model; *C. sinensis* (Bailing-High, BH, 5 g/kg ), PD1 inhibitor (PD1, 2 mg/kg); PD1 inhibitor combined with a low dose of *C. sinensis* (2 mg/kg of PD1 inhibitor + 1 *g*/kg of *C. sinensis*, PD1 + BL); PD1 inhibitor combined with a high dose of *C. sinensis* (2 mg/kg of PD1 inhibitor + 5 g/kg of *C. sinensis*, PD1 + BH).

A *C. sinensis* solution was prepared in 0.5% sodium carboxymethyl cellulose (10 mL/kg). The PD1 inhibitor (20 mL/kg) was injected intraperitoneally. Distilled water was used as the treatment for mice in the control and model groups. Two weeks later, blood and lung tumor tissues were collected for biochemical analysis, pathologic evaluation, immunohistochemistry, transcriptomics sequencing, proteomics, and Western blot.

### Histologic observations of the lung

Mouse lung tissues were obtained from each group and placed in 4% paraformaldehyde and fixed overnight. Paraffin embedding was performed by ethanol dehydration, then the tissue sections were sliced, dewaxed, and rehydrated. For histopathological analysis, tissue sections were stained using hematoxylin and eosin (H&E) and the slides were scanned for examination.

### Biochemical analysis in serum by ELISA

ELISA kits for, interleukin (IL)-6, IL-10, tumor necrosis factor-α (TNF-α), superoxide dismutase (SOD), glutathione peroxidase (GSH-Px), malondialdehyde (MDA), erythropoietin (EPO), and granulocyte-macrophage colony-stimulating factor (GM-CSF) were obtained from Shanghai Enzyme-linked Biotechnology (ml002095, ml063159, ml037873, ml016824, ml037757, ml643059, ml002210, and ml037645, respectively; Shanghai, China). Biochemical analyses of sera for the above factors were performed in accordance with instructions supplied by the manufacturer.

### Transcriptomics sequencing and data analysis

RNA was extracted from lung tissue from mice (*n* = 3 per group), an Illumina TruSeq RNA library was constructed, and Illumina NovaSeq 6000 (San Diego, CA, USA) was used for sequencing. Change was defined by setting the cut-off value at 2-fold, and the statistically significant threshold was set at *P* < 0.05 for differentially-expressed gene screening. Finally, gene ontology (GO) function enrichment analysis and gene set enrichment analysis (GSEA) were conducted using the R programming language.

### Proteome detection and mass spectrometry

Proteins were extracted from mouse lung tissues of the control, model, and BH groups. Briefly, 0.1 *g* of lung tissue from each sample were obtained and rapidly ground by adding appropriate lysis solution (RIPA + 1X protease inhibitor cocktail) for complete lysis of proteins, then the supernatant was obtained after centrifugation (15,000 g for 20 min at 4 ℃). After quantification, 100 µg/100 µL of protein from each group were incubated in 5 mM dithiothreitol for 30 min at 37 ℃ in the dark to allow chemical reduction of disulfide bonds. Next, proteins that had been alkylated with 20 mM iodoacetamide were placed in the dark for 30 min. Samples were washed three times by the following steps: 400 µL of methanol (pre-chilled at − 80 ℃); 100 µL of methylene chloride; and 200 µL of hyper-pure water. The supernatant was discarded and 500 µL of pre-chilled methanol was added to the pellet obtained after centrifugation (15,000 *g* for 3 min at 4 °C). Then, proteins were dissolved by adding 200 µL of buffer solution of 200 mM 4-(2-hydroxyethyl)-1-piperazine propane sulfonic acid at pH 8.5 to the protein precipitate, and further digested by trypsin at 37 ℃ for 17 h (protein: enzyme = 100 µg: 1 µg). Samples were desalted using a commercial C18 column (Waters, Milford, MA, USA), dissolved in 50 µL of 0.1% formic acid (FA) and centrifuged (15, 000 x *g* for 30 min). Supernatant aliquots (10 µL) were analyzed via liquid chromatography with tandem mass spectrometry for protein identification.

### Database search and proteomic analysis

The Xcalibur analysis system (Thermo Fisher, Waltham, MA, USA) was used to collect MS data. Protein identification was performed using Proteome Discoverer V2.4 aligned to the NCBInr (http://www.ncbi.nlm.nih.gov/) and UniProt databases (http://www.uniprot.org/) with the Sequence HT algorithm. Differentially-expressed proteins (DEPs) were screened based on the following standards: upregulated, FC > 1.5 and *P* < 0.05; downregulated, FC < 0.67 and *P* < 0.05. Then, the DEPs were analyzed by GSEA.

### Immunohistochemical staining

The immunohistochemical staining protocol comprised a number of steps, as follows. First, mouse lung tissue from each group was obtained and placed in 4% paraformaldehyde for a 3-h fixation before embedding in paraffin for 24 h at 60 ℃. The paraffin specimens were then dewaxed using xylene and subsequently dehydrated in an ethanol gradient. The dewaxed sections were submerged in water, then in citrate buffer (C1032; Solarbio, Beijing, China) and heated in a microwave oven to allow antigen repair. Each section was then flushed with PBS 3 times and sealed with 3% hydrogen peroxide and 5% bovine serum albumin for 10 min and 1 h, respectively. Next, rabbit anti-mouse Ki-67 (9440 S; CST, Danvers, MA, USA) and rabbit anti-mouse VEGFA polyclonal antibodies (19003-1-AP; Proteintech, Wuhan, China) were added to the samples, which were then incubated overnight at 4 ℃ in a refrigerator. On the following day, samples were washed three times with PBS. Then HRP-labeled goat anti-rabbit IgG (PR30009; Proteintech; 100 drops) was added to each slide, followed by incubation at 25 ℃ for 1 h. DAB staining (DA1010; Solarbio), hematoxylin staining, gradient ethanol dehydration, and neutral gum sealing were then performed.

### Western blot analysis

Western blot analysis was performed according to a previously described protocol [24]. In brief, lung tissue proteins were extracted from each group and the level was measured using a BCA kit (Beyotime, Beijing, China) and adjusted to a final concentration of 1 µg/µL. Next, 5× loading buffer solution was added to the protein at a ratio of 1:4 ratio and heated for denaturation at 96 ℃ for 10 min. A 30-µg protein from each group was separated via SDS-PAGE and then transferred to a PVDF membrane. After a 1-h sealing step with 5% bovine serum albumin (ST2254; Beyotime) at room temperature, the membrane was incubated with primary antibodies overnight in a refrigerator at 4 ℃. The primary antibodies included anti-RhoA (1:1000; Bioss, Beijing, China), anti-raf1 (1:1000; Bioss), and anti-c-fos (1:1000; Bioss). The next day, the membrane was cleaned three times with TBST solution (10 min per time) and incubated with a HRP-linked goat anti-rabbit/mouse IgG, antibody (1:5000; Bioss) at room temperature for 1 h. The membrane was washed a further three times in TBST buffer (10 min per time). Finally, ECL luminescent liquid was added for visualization. The above experiments were repeated three times and image J software was used for calculations.

### Routine blood testing

Blood samples were collected from anesthetized mice and stored at 4 ℃. A SYSMEX XN-1000 V instrument (Kobe, Japan) was used to measure platelets (PLTs), white blood cells (WBCs), red blood cells (RBCs), and hemoglobin (HGB).

### Flow cytometric analysis of immune cells

Flow cytometry was conducted using a Beckman Coulter counter (CytoFLEX, Brea, CA, USA) in accordance with the instructions supplied by the manufacturer. Blood samples were collected in heparin sodium anticoagulant tubes with red blood cell lysis buffer. Then, after splitting on ice in the dark for 15 min, samples were centrifuged (4 ℃ at 400 × *g* for 5 min) and incubated with antibodies (4 ℃ for 30 min) to separate DCs, and NK cells, and CD4^+^ and CD8^+^ T cells.

## Results

### ***C. sinensis*** alleviated NSCLC in mice

**Fig. 1:**
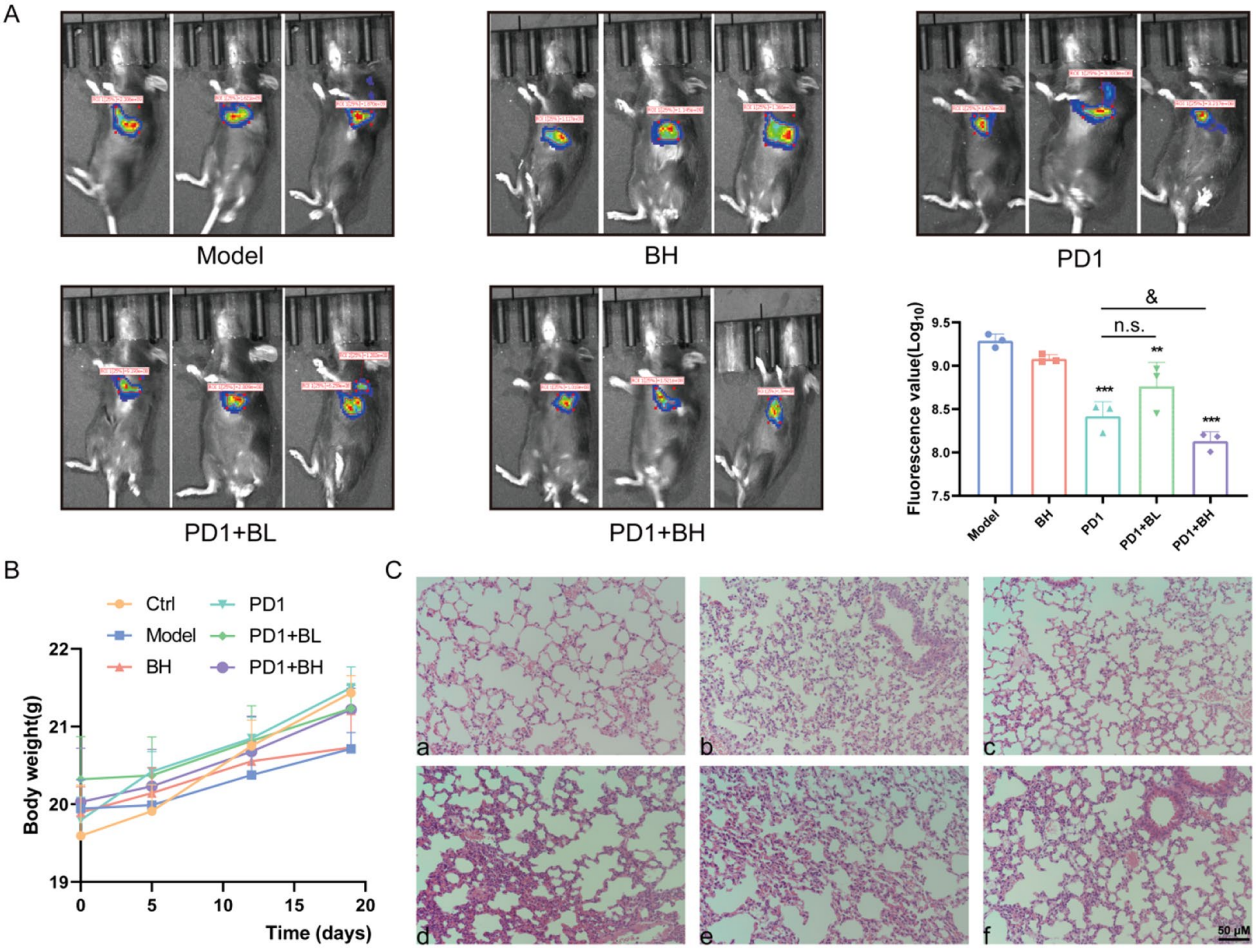
*Cordyceps sinensis* inhibited lung cancer in mice with NSCLC. **A** Fluorescence photographs of mice were obtained 2 weeks after administration (*n* = 3; **P < 0.01 and ***P < 0.001 versus the model; ^&^*P* < 0.05, vs. PD1). **B** Body weight for mice in the different treatment groups (*n* = 6). **C** Microscopic morphology of the lung tumor tissues in each group (HE, 20×). **a** Control group (Ctrl); **b** Model group (Model); **c** *C. sinensis* group (5 *g*/kg, BH); **d** 2 mg/kg PD1 inhibitor group (PD1); **e** 2 mg/kg PD1 inhibitor + 1 *g*/kg *C. sinensis* group (PD1 + BL); **f** 2 mg/kg PD1 inhibitor + 5 *g*/kg *C. sinensis* group (PD1 + BH).

The anti-tumor effect of *C. sinensis* against NSCLC was evaluated in mice. After a consecutive gavage administration for 2 weeks, we collected lung tissue images from all mice in the different groups (*n* = 3) using small animal live imaging. Figure 1A shows that the fluorescence for mice in the model group was 9.29 × 10^9^, whereas the fluorescence of mice in the administration groups was significantly decreased. A high dose of *C. sinensis* combined with PD1 tended to show a better effect, with a statistical difference in comparison with the model group. Compared to PD1 inhibitor, the effect of PD1 was more pronounced in reversing the above changes when used in combination with a high dose of *C. sinensis* (*P* < 0.05). This finding demonstrated that *C. sinensis* suppressed mouse tumor growth in the orthotopic lung cancer model. In addition, neither the single use of *C. sinensis* or PD1 nor the combination significantly changed the body weight of mice (Fig. 1B), indicating the safety of drugs. The HE staining results suggested that the bronchial and alveolar structures of healthy mice were complete. The alveoli of mice with NSCLC were severely damaged with structural disorders and some exhibited alveolar atresia, fissures, and collapse. In contrast to the model group, mice in the BH and PD1 groups showed less damage. Similarly, the above phenomena were also ameliorated and alleviated by combination treatments (PD1 + BL and PD1 + BH), as shown in Fig. 1C.

### ***C. sinensis*** markedly ameliorated serum inflammatory cytokines and oxidative stress indicators in mice with NSCLC

**Fig. 2:**
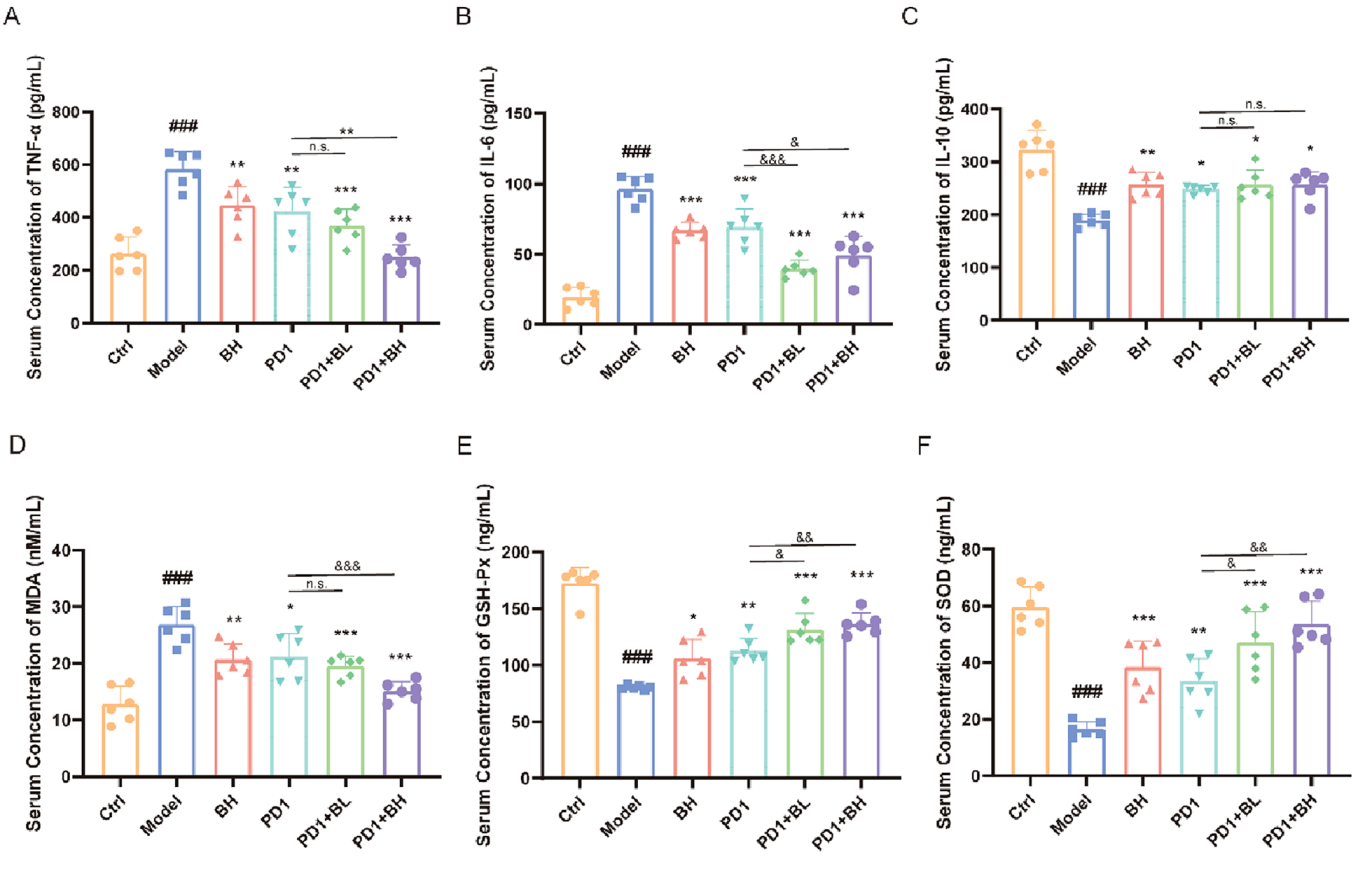
Influence of *Cordyceps sinensis* on inflammatory cytokines and indicators of oxidative stress in serum from mice with NSCLC.** A**–**C** Serum TNF-α, IL-6, and IL-10 concentrations in different groups. **D**–**F** Serum oxidative stress indicator (MDA, GSH-Px, and SOD) production in mice. Results are expressed as mean ± SD for *n* = 6 mice in each group. ^###^*P* < 0.001 vs. control; *P < 0.05, **P < 0.01, ***P < 0.001 vs. model; ^&^*P* < 0.05, ^&&^*P* < 0.01, ^&&&^*P* < 0.001, vs. PD1

Lung cancer cells recruit monocytes, neutrophils, and other WBCs by secreting various mediators, thereby producing a series of inflammatory factors [25]. Reactive oxygen species (ROS), the oxidative product of cell metabolism, damages lipid, nucleic acid, and protein function, breaks the oxidative balance, induces oxidative stress, and promotes inflammation, all of which lead to various diseases [26, 27]. As shown in Fig. 2, in contrast to the control mice, serum TNF-α, IL-6, and MDA concentrations in mice with NSCLC were significantly increased, whereas IL-10, SOD, and GSH-Px concentrations were lower. In comparison to the model group, single use of *C. sinensis* and *C. sinensis* in combination with PD1 showed statistical differences in reversing the above changes. Moreover, the effect of PD1 was more pronounced in reversing the above changes when used in combination with *C. sinensis*.

### ***C. sinensis*** had a regulatory effect on inflammation and immune-related genes in NSCLC mice

**Fig. 3:**
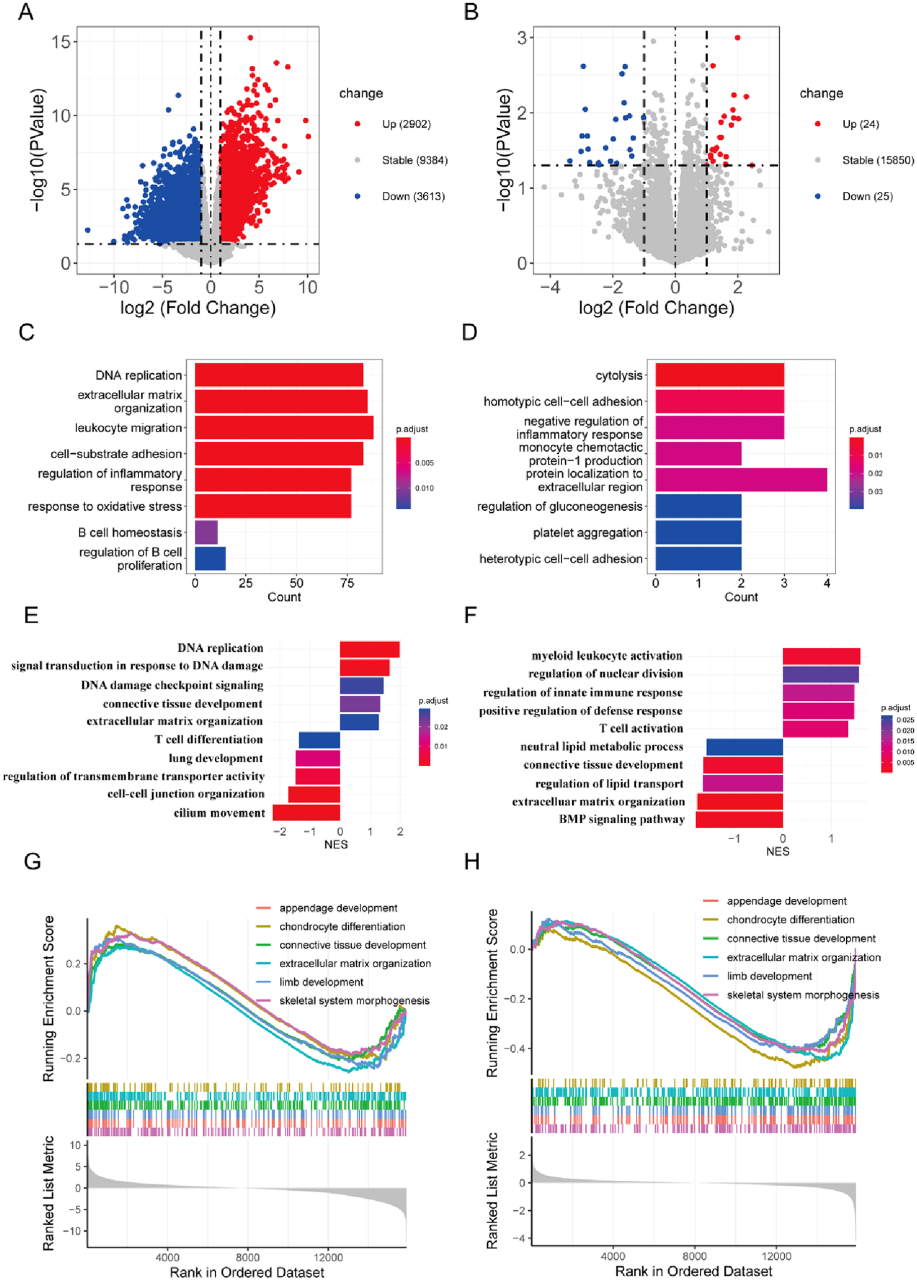
DEG analysis of *C. sinensis* against NSCLC. Volcano maps showing DEGs for (**A**) mice with NSCLC versus healthy mice and (**B**) mice with NSCLC versus BH mice. **C**, **D** GO (BP) enrichment results of DEGs in the model/control and BH/model groups. **E**–**H** GSEA enrichment results of DEGs in the model/control and BH/model groups

To screen out key genes related to *C. sinensis* administration against NSCLC, we carried out RNA sequencing (RNA-Seq) on samples of murine lung cancer tissue from animals in the control, model, and BH groups. As shown in Fig. 3A, B (the volcano map), there were different amounts of DEGs among the control, model, and BH groups based on the transcriptomic data. Comparison to the control group revealed that 6515 DEGs were significantly different in the model group, with 2902 DEGs upregulated and 3613 DEGs downregulated. Comparison to the model group revealed that 49 DEGs were markedly different in the BH group (including 24 upregulated and 25 downregulated DEGs). The DEGs upregulated in the model group in comparison to the control group were mainly enriched in pathways with biological processes, including leukocyte migration, oxidative stress responses, and regulation of the proliferation of B cells (Fig. 3C). The downregulated DEGs of *C. sinensis* administration against NSCLC were mainly enriched in pathways with biological processes, including production of monocyte chemotactic protein-1 and negative regulation of the inflammatory response (Fig. 3D). Moreover, we analyzed the DEGs between different groups through GSEA enrichment and observed high enrichment scores for the signaling pathways associated with the differentiation and activation of T cells (Fig. 3E–H).

### ***C. sinensis*** effectively regulated inflammation and immune-related proteins in the lung tissues of mice with NSCLC

**Fig. 4:**
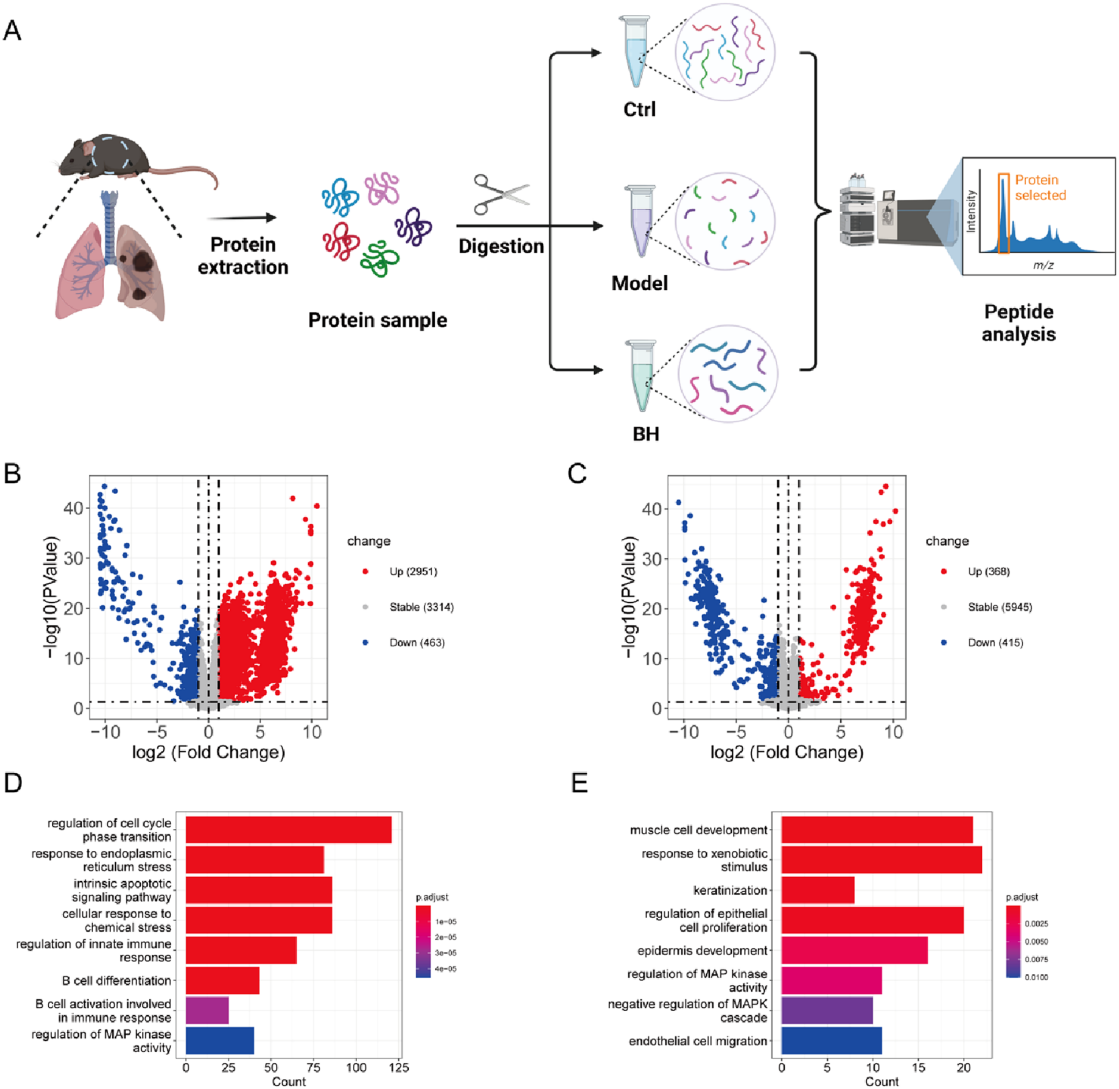
DEP analysis of *Cordyceps sinensis* against NSCLC. **A** Flow chart of proteomics analysis. Volcano maps showing DEPs for **B** the model versus control groups and **C** the BH versus model groups. **D**, **E** GO (BP) enrichment result of DEPs in the model/control and BH/model groups

Various quantification proteomic experiments enable the accurate identification of differentially-expressed proteins (DEPs) of normal versus tumor tissues [28]. We adopted label-free quantification technology to detect DEPs in the lung tissues of the mice in different groups. The protein files of different groups were label-free quantified (Fig. 4A). As shown in Fig. 4B, C, we identified 3414 DEPs between mice with NSCLC and healthy mice (including 2951 upregulated and 463 downregulated DEPs). In contrast to the model group, 783 DEPs were markedly different in the BH group (including 368 upregulated and 415 downregulated DEPs). The DEP biological functions, metabolic pathways, and signal transduction pathways were analyzed by GO enrichment. We showed that the upregulated DEPs in the model versus control groups were mainly enriched in pathways with biological processes, including B cell differentiation, B cell activation involved in immune responses, and regulation of MAP kinase activity (Fig. 4D). The downregulated DEPs of BH administration against NSCLC were mainly enriched in pathways with biological processes, including upregulation of mitogen-activated protein (MAP) kinase activity and downregulation of the MAP kinase (MAPK) cascade (Fig. 4E). B cells have an antigen-presenting function in the immune system, whereby antigen is presented to T cells via the B-cell receptor (BCR) in an MHC-restricted manner to activate T cell immune responses and ultimately kill cancer cells [29]. At the same time, the p38 MAPK pathway is involved in control of cell proliferation via targets that are mainly downstream of the BCR [30]. The Ras-MAPK cascade is manifested in different cell types and regulates biological functions [31]. Accumulating evidence has shown that various types of malignant tumors, including NSCLC, are closely related to MAPK signaling deregulation [32, 33]. Our findings also suggested that *C. sinensis* exerted an anti-cancer effect against NSCLC mice by suppressing MAPK pathway-mediated tumor cell proliferation.

### ***C. sinensis*** markedly inhibited the MAPK signaling pathway in mice with NSCLC

**Fig. 5:**
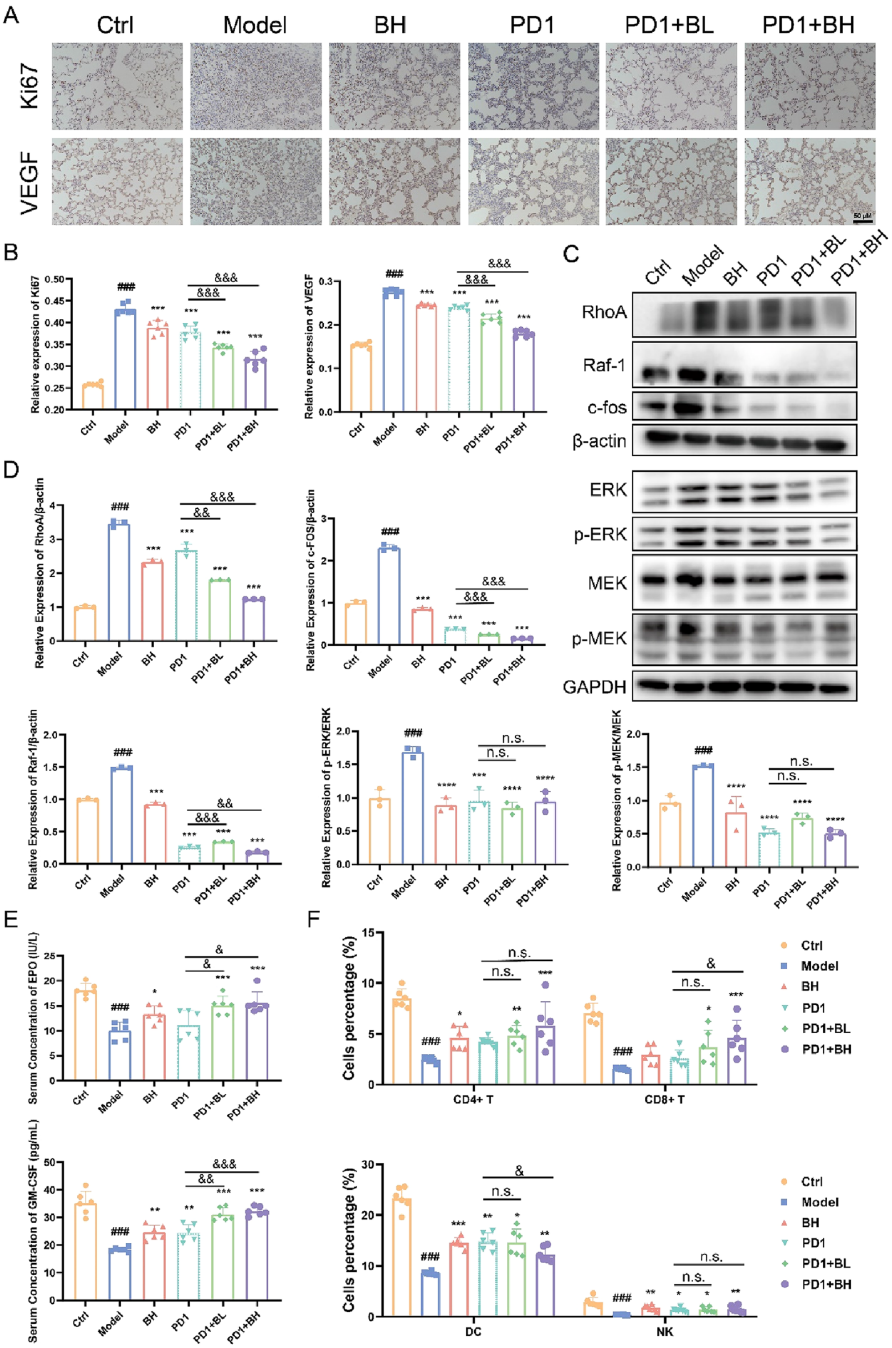
Influence of *Cordyceps sinensis* on the levels of MAPK protein expression signaling pathway protein expression in mice with NSCLC. **A**, **B** Immunohistochemical staining (20×) was performed using different types of antibodies. Tumor expression of Ki67 and VEGF was analyzed via immunohistochemistry. Ki-67 is a protein marker of proliferation encoded by the MKI67 gene. VEGF, vascular endothelial growth factor. **C**, **D** Protein level analysis by Western blot in tumor tissues of key targets, including RhoA, Raf-1, c-fos, ERK1/2, MEK1/2 and their phosphorylated forms in the MAPK signaling pathway, respectively. **E** The serum hematopoietic growth factor (EPO and GM-CSF) concentration in mice from all groups. **F** Proportions of CD4^+^T cells, CD8^+^T cells, DCs, and NK cells in different groups. All values are expressed as the mean ± SD with *n* = 6 in each group. ^###^*P* < 0.001, vs. Control; *P < 0.05, **P < 0.01, ***P < 0.001, vs. Model; ^&^*P* < 0.05, ^&&^*P* < 0.01, ^&&&^*P* < 0.001, vs. PD1

According to a previous report, VEGF induces the formation of microvessels during the development of NSCLC and further mediates NSCLC occurrence and metastasis [34]. In addition, the Ki67 labeling index, a typical marker used to measure the proliferation of tumors, is routinely used to detect a variety of cancers [35]. Levels of VEGF and Ki67 protein expression in tumor tissues from the different treatment groups were analyzed via immunohistochemistry to verify the results of transcriptome and proteome analyses in our study (Fig. 5A, B). VEGF and Ki67 protein levels detected in the lung tissues of NSCLC mice were upregulated in contrast to the levels of VEGF and Ki67 protein in lung tissue from healthy mice. Use of *C. sinensis* alone and the combination of *C. sinensis* with PD1 statistically decreased the protein concentration (*P* < 0.001). And the simultaneous use of the two drugs reduced their protein content more significantly (*P* < 0.001). The Rho family acts as a regulatory factor to mediate cell migration, among which RhoA is a key member involved in regulating tumor cell invasion and metastasis by affecting the cellular cytoskeleton [36, 37]. As reported, RhoA activates Raf, which is an upstream molecule of the MAPK pathway [38]. MEK is downstream of the RAS-RAF-MEK-ERK pathway (ERK signaling), which is one of the MAPK pathways, and its aberrant activation associates with various tumorigenic diseases, including NSCLC [39, 40]. In the current study we investigated the influence of *C. sinensis* on the levels of key protein expression in the MAPK signaling pathway by Western blot. As shown in Fig. 5C, D, the single use of *C. sinensis* and the combination of *C. sinensis* with PD1 markedly downregulated RhoA, Raf-1, and c-fos expression, which are related to cell migration and invasion, and the combination group was superior to single therapy of *C. sinensis* or PD1 (*P* < 0.01). Furthermore, total protein quantity of ERK1/2 and MEK1/2 did not change significantly, but their phosphorylated forms were significantly downregulated with the single use of *C. sinensis* or PD1 or the combination.

To further evaluate the influence of *C. sinensis* on immune function of mice with NSCLC, the levels of hematopoietic growth factors (EPO and GM-CSF) were determined. As shown in Fig. 5E, after 5 days of modeling, the levels of hematopoietic factors (EPO and GM-CSF) had significantly decreased in the model group (*P* < 0.01). At 14 days, the EPO and GM-CSF levels of each administration group were markedly increased. Percentage levels of CD4^+^ and CD8^+^ lymphocytes were significantly lower in the model group than in the control group (*P* < 0.001); however, percentage levels of CD4^+^ and CD8^+^ T cells that decreased after model treatment increased in the BH and PD1 groups (*P* > 0.05). Interestingly, the combination group effectively reversed the above phenomenon (Fig. 5F). NK cells recognize and eliminate stress cells during cancer, whereas DCs are also critical during the antitumor process by regulating the initiation, development, and maintenance of the immune response [41]. As shown in Fig. 5F, our findings indicated that levels of NK cells and DCs were significantly lower in the model group than in the control group (*P* < 0.001). In agreement with the above transcriptomics and proteomics results, the administration groups reversed the above results and further demonstrated that *C. sinensis* produced a marked effect on mice with NSCLC by activating immune cells. Compared with the single therapy of *C. sinensis* or PD1, their combination exerted better anti-tumor effect. Taken together, these results indicate that *C. sinensis* probably exerts a protective effect against NSCLC by suppressing the expression of the RhoA gene, then recruiting immune cells, enhancing immune function, and inhibiting lung cancer via the MAPK pathway (Additional file 1, Figure S2).

## Discussion

Among the lung cancer histological subtypes, non-small cell lung cancer (NSCLC) is the most common [42]. Recently, although the emergence of new targeted drugs has provided more choices for patients, the high price and tolerance and accompanying NSCLC patient unfavorable survival rate limit use [43, 44]. With accurate therapeutic effects and a minimal side-effect profile, traditional Chinese medicine (TCM) has long been used as a treatment for cancer. *C. sinensis*, a widely-used and well known traditional Chinese medicine, was officially classified as a drug in 1964 in the Chinese Pharmacopoeia [45]. As the most studied and applied specie among Cordyceps, *C. sinensis* has already been reported exerting pharmacological activities like antioxidant, anti-cancer, antihyperlipidemic, anti-diabetic, anti-fatigue, anti-aging, anti-depressant, and kidney protection, which presents huge potential for active medical transformation. C. *sinensis* powder has been reported to be effective in the inhibition of NSCLC both in the single use and the combination with other drugs in previous studies [46, 47]. However, the characteristics of pharmacological effects and molecular mechanisms when C. *sinensis* was used in the combination with PD-1 inhibitor haven’t been clarified.

Based on the above, we chose an orthotopic cancer model with the subcutaneous injection of lung cancer cells to mimic NSCLC. We successfully constructed an NSCLC mouse model. The alveoli of mice were severely damaged and structurally disrupted, with some alveoli being blocked, ruptured, and collapsed. In addition, a significant increase in the number of lung cancer cells is evident in the in vivo images. Using the NSCLC model, we showed that the anti-inflammatory activities of *C. sinensis* by significantly inhibiting inflammatory cytokines and oxidative stress indicators in mouse serum. Our study provided direct preclinical evidence that *C. sinensis* inhibited tumor growth, alleviated the degree of alveolar damage, and had an anti-inflammatory role in mice with NSCLC.

Subsequently, we explored the key genes of *C. sinensis* acting on NSCLC in mice through transcriptomics sequencing to reveal the molecular mechanism underlying the development of lung cancer. Our results for transcriptome sequencing prompted us to focus on the RhoA gene, for which a role has highlighted in tumor cell invasion and metastasis [37] in malignancies such as breast cancer [48], nasopharyngeal carcinoma [49], and NSCLC [50]. Cytokines and chemokines are critical in regulating T-cell recruitment and the overall cellular composition of the tumor microenvironment [51–53]. Activated RhoA leads to the formation of stress fibers within cells and expression of chemokines, cytokines, and growth factors [54, 55]. The important role of immune function in the incidence and development of lung cancer is well established [56–58]. As reported, fermented cordyceps powder reduces radiation-induced bone marrow suppression, increases the percentage of peripheral blood leukocytes, improves chemotherapy tolerance, and promotes spleen cell proliferation, NK cell activity, and T-helper cytokine secretion in mice with impaired immune function [59, 60]. Based on the GO (BP) enrichment and GSEA enrichment of DEGs, transcriptome data confirmed that *C. sinensis* promoted T cell differentiation and activation, and negatively regulated the inflammatory response. T cells are known to regulate cellular immunity and play an essential role in successful treatment of lung cancer. T cells recognize tumor antigens (proteins expressed only by tumor cells that are induced by genetic mutations in these cells) and trigger strong anti-tumor immune effects, making T cells important targets for tumor treatment.

To systematically clarify the mechanism underlying *C. sinensis* action on NSCLC, we further analyzed the protein expression in lung tumor tissues via proteomics. According to the proteomics results, the DEPs of the BH versus model groups were characterized by significant upregulation of MAP kinase activity, and downregulation of MAPK cascade pathways. As described, tumor‑associated macrophages (TAMs) promote the proliferation and invasion of tumor cells by activating the MAPK signaling pathway [61]. As an autocrine growth factor for NSCLC cells, VEGF has a crucial role in angiogenesis and promotes lung tumor growth [62, 63]. As a marker of tumor cell proliferation, the Ki-67 index increases to higher levels with tumor development [64]. Herein, expression levels of VEGF and Ki67 at the protein level in tumor tissues of different treatment groups were detected by immunohistochemistry to indicate decelerating proliferation of tumors in NSCLC mice. Notably, RhoA regulates immune cell differentiation and function, recruits innate immune cells (including neutrophils and macrophages), enhances the antigen presentation ability of immune cells, forms immune synapses, and improves the tumor microenvironment [65–67]. Liu et al. concluded that KLHL17 upregulation in NSCLC promotes tumor cell proliferation and migration via an increase in RhoA expression, as well as activation of the pathway responsible for Ras/MAPK signaling [68]. Additionally, RhoA Raf is activated by RhoA upstream of the MAPK pathway [38, 69]. Western blot results showed that *C. sinensis* treatment downregulated RhoA, Raf-1, c-fos, phosphorylated ERK1/2 and MEK1/2 in lung tissues of NSCLC mice. Moreover, we showed that EPO and GM-CSF in each administration group were markedly increased. Interestingly, after 14 days of treatment with *C. sinensis*, the proportion of immune cells significantly increased, indicating that *C. sinensis* mitigated the immune dysfunction due to lung cancer. This finding reflects an important immunostimulatory mechanism in the inhibition of lung cancer cell growth by *C. sinensis*. The use of *C. sinensis* has been documented in drugs for respiratory infections, as well as promoting activation of the immune response [70]. Based on previous studies, the active ingredients from *C. sinensis* were mostly recognized by Toll-like receptors and C-type lectin receptors during initiation of immunomodulation and more importantly, the following intracellular signaling initiated from the receptors. *C. sinensis* can likely defense cancers by recruiting and augmenting immune cells such as natural killer cells and macrophages [71–74]. Although the exact mechanism remains unclear, multiple researches have reported *C. sinensis* exerted the effect of recruiting immune cells, such as limiting the expression of cytokines like IL-2 and TNF-α [75], inhibiting MAPK pathways [76], and inducing redistributions of peripheral mononuclear T lymphocytes [77]. Hence, *C. sinensis* likely exerted a protective effect against NSCLC by suppressing the RhoA gene, then recruiting immune cells, enhancing immune function, and inhibiting lung cancer via the MAPK pathway.

## Conclusion

To summarize, we successfully constructed an NSCLC mice model and determined the potential mechanism and target of *C. sinensis* by which immune cells are recruited via the MAPK signaling pathway and ultimately inhibit the proliferation of lung cancer cells through a combination of transcriptomics, proteomics, and experimental verification. Importantly, our study elucidated the action of *C. sinensis* in alleviating NSCLC, which will likely contribute to clinical application in the treatment of NSCLC.

### Supplementary Information

**Additional file 1:Fig. S1.** Quality control data of *Cordyceps sinensis* powder (Bailing Capsule). **Fig. S2.** *Cordyceps sinensis* likely exerted a protective effect against NSCLC via the MAPK pathway.

## Data Availability

The datasets used and analyzed during the current study are available from the corresponding author on reasonable request.

## Abbreviations

BCA: Bicinchoninic acid
CMC-Na: CarboxymethylCellulose
DEGs: Differentially expressed genes
DEPs: Differentially expressed proteins
DMEM: Dulbecco’s modified eagle medium
ECL: Enhanced chemiluminescence
EPO: Erythropoietin
GM-CSF: Granulocyte-macrophage colony stimulating factor
GO: Gene ontology
GSH-Px: Glutathione peroxidase
HE: Hematoxylin-eosin; IL-6: Interleukin-6
IL-10: Interleukin-10
GSEA: Gene set enrichment analysis
MAPK: Mitogen-activated protein kinase
MDA: Malondialdehyde
NSCLC: Non-small cell lung cancer
PD1: Programmed death-1
PVDF: Polyvinylidene fluoride
RIPA: Radio immunoprecipitation assay
SOD: Superoxide dismutase
TNF-α: Tumor necrosis factor-α
VEGFA: Vascular Endothelial Growth Factor A
