# Supplementary material for: Cordyceps sinensis relieves non-small cell lung cancer by inhibiting the MAPK pathway
Source: Chin Med. 2024 Mar 25;19:54. doi: 10.1186/s13020-024-00895-0 (PMC10962170; doi:10.1186/s13020-024-00895-0)
Supplement: Supplementary file 1 — Additional file 1:Fig. S1. Quality control data of Cordyceps sinensis powder (Bailing Capsule). Fig. S2. Cordyceps sinensis likely exerted a protective effect against NSCLC via the MAPK pathway. [file 13020_2024_895_MOESM1_ESM.pdf]

Supplementary material

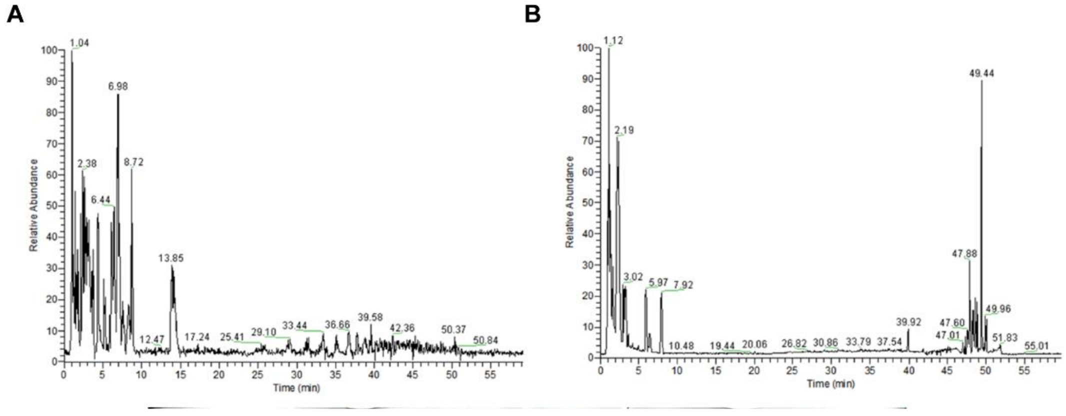

Figure S1. Quality control data of Cordyceps *sinensis* powder (Bailing Capsule). (A-B) TIC diagram of the Cordyceps *sinensis* powder in Bailing Capsule UHPLC-LTQ-Orbitrap-MS, and (A) for positive mode, (B) for negative mode.

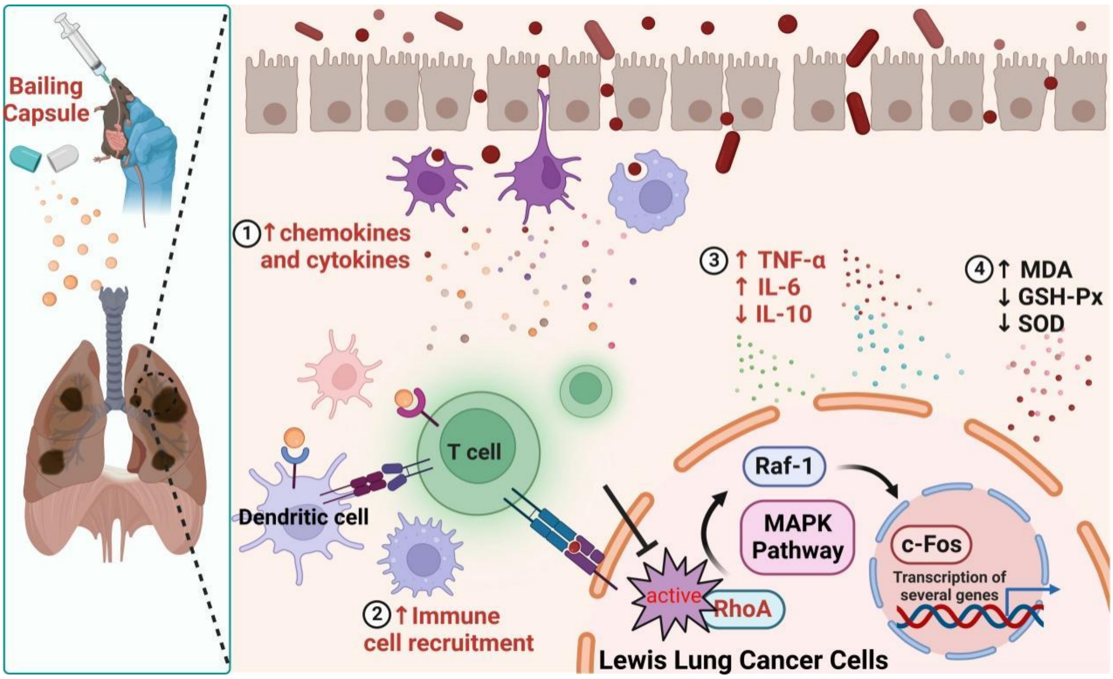

Figure S2 *Cordyceps sinensis* likely exerted a protective effect against NSCLC by suppressing the RhoA gene, then recruiting immune cells, enhancing immune function, and inhibiting lung cancer via the MAPK pathway
